# The Science and Art of Housekeeping

**Published:** 1888-08-11

**Authors:** 


### The Science and Art of Housekeeping.

In the days of our grandmothers housewifery was learned with great thoroughness in the home kitchen. In the days of our mothers the instruction, though less constant and prolonged, though the preserving and pickling was a less serious part of life's duties, though the making of light and flaky pastry had ceased to be the very crown of womanly accomplishments—for the making of wax flowers and the singing of sentimental ballads had set up a certain rivalry—still, in the days of our mothers a very fair knowledge of domestic duties was gained between the time of leaving school and of going to that "house of her own," which is every girl's dream and ideal. But in our own days—woe betide us! We had learned Euclid at school, and physiology—with the exception of the machinery of digestion, which our head mistress, bless her sweet shy spinsterhood, didn't consider quite delicate. But when we came home "for good" we found that cook got very cross at our "messing about," if we wanted to try the making of a cake or a pudding, the recipe for which we had got out of the *Girl's Own Paper*, or the *Ladies' Treasury*, and our mothers said with a smile, when we expressed a desire to study any of the arts of the household, that we should "learn soon enough." Wherefore we began to think that counterpoint, and china-painting, and lawn tennis formed the business of life; and cooking, and mending, and the mysterious power of vision that can "see dust" only its accidents.

Further experience convinced us that our estimate of life was somewhat mistaken. The lover who had been our partner in many a well-contested sett, or whom we had won by reading difficult accompaniments at first sight, developed into the husband who wanted his dinner; and, alas! we could neither cook it ourselves nor instruct our servants how to cook it for him. They, the inexperienced servants who fall to the lot of impecunious young married couples, had spent the greater part of their lives at board schools, and were well nigh as inexperienced as their mistresses. Then it dawned upon us that the educational system that failed to give girls, the majority of whom were destined to be either servants or mistresses of households, any instruction in housekeeping was rather defective. Intellectual development is a very good thing; but is not the intellectual development, which either unfits people for, or leaves them uninstructed in the work which is to fall to them as daily duty throughout life, mistaken, or else incomplete?

Things are mending for both mistresses and servants. There has lately been instituted a national association among the many that claim that far-reaching appellation, which is not unworthy of the name—the "National Housewifery Association." The intention of this is avowedly to instruct girls of the middle-class in those domestic arts which are omitted from the general school curriculum, while giving them at the same time a sound but simple English education. Music and French may be learned by those who so desire; but these are "extras," while cooking, scrubbing, and laundry work are integral parts of the course. Miss Headdon, the principal of the association, is desirous, also, of introducing classes for dressmaking, type setting, and other branches of technical instruction for those girls whom fate promises to call into the more open fields of the world's life; those who have to make money, as well as to save it. But it is the domestic work which is the special feature of the school which Miss Headdon has established at the Villa-Bullo at Newnham-on-Severn. This is divided into three sections, viz., a domestic kinder-garten for the little ones, a junior course of practical housework for those a little more advanced, and a senior course for imparting a scientific knowledge of housewifery, hygiene, and sanitary subjects generally for the elder girls. So, young women educated at the Villa-Bullo will begin by "playing at houses," as all little girls love to do, and go on gradually to larger practice and comprehension of the duties which in all probability will occupy them through life. Doubtless all will not turn out equally well; the housekeeper, like the poet, is born, not made; but the housekeepers, like the poet will improve natural talent by study and practice, and the least successful of Miss Headdon's pupils will never disgrace herself in her cook's eyes as did poor young Mrs. Jerningham, as she narrates in her journal:—

"I said, 'A leg of lamb you'll get;'
Said she, 'It's not in season yet;'
Then turning somewhere for relief,
I said, 'Well, get a leg of beef.'
At that she looked me in the face,
She made me feel the full disgrace.
At last I said, 'Get anything,'
And ran upstairs to play and sing.
I hope we'll have some dinner, though;
Or John might be displeased you know."

Poor John!—poor little wife, set to perform duties in which she has had no training! We are very sorry for her, but the sooner she and her sisters disappear from the face of the earth the better. So may all good attend the National Housewifery Association, which is determined on a beneficent crusade of extermination.

But the new must not make us forget the old, and there is an institution in London, which for nearly two hundred years has been bringing up young girls to be domestic servants. The Ladies' Charity School, Powis Gardens, Notting Hill, was opened in 1702, for the purpose of training the daughters of respectable poor parents, not necessarily orphans, to be domestic servants. There they are kept from the ages of eight to fifteen, receive a sufficient education, and are trained in domestic work. While it was comparatively young the school met with the approval of gruff, shrewd, kindly old Dr. Johnson, who annually subscribed two guineas to its funds as long as he lived, and at his death bequeathed to it his "teaspoons," now carefully kept as a relic. He founded on it his once popular story of "Betty Broom," and the girls at the school still wear the quaint, pretty "Betty Broom" costume, a short dress of bluish-grey, with a high cap, apron, and shoulder cape of stiff and spotless white, to which on gala days a pair of long white mittens is added. Mrs. Thrale, Dr. Johnson's lively and amiable friend, was also a subscriber to the institution, which is now under the patronage of the Queen, and has been since her Majesty was the Princess Victoria of Kent. Before her, however, the Princess Charlotte, whose untimely death plunged the nation into grief, was its first royal patroness.

The records kept of the girls after they leave school show how efficient is the training, and also indicate that, though domestic service is the general goal to which the pupils are destined, they are not compelled to seek it if they show more inclination to other work. The general record is, "has received long service reward," on being five, seven, or nine years in one situation, with occasionally the appended words, telling of the greatest record of all, "married well." But we find that one ex-pupil went into the telegraph office, and is now a post-mistress, another is a nurse at Guy's, a third was till her marriage head mistress in a national school, and a fourth head dressmaker in a large London shop. These are honourable records, and should do much to ensure the means of continued and increasing prosperity to the school, which gives poor and often friendless girls the means of becoming useful and prosperous members of society. Meanwhile, with these two institutions training girls in what always has been and what always will be woman's work, the young men who say they won't marry because girls don't know anything of house-keeping, and the mistresses who complain that their servants don't know their work or won't do it, may look forward to better times.

